# Long‐term renal functional outcomes following ureteroureterostomy performed during multi‐organ resection for non‐urothelial cancers

**DOI:** 10.1002/bco2.88

**Published:** 2021-05-05

**Authors:** Phillip W. Pisters, Weranja Ranasinghe, Wei Wei, Christopher G. Wood, Surena F. Matin, John F. Ward, Louis L. Pisters

**Affiliations:** ^1^ Department of Urology University of Texas M.D. Anderson Cancer Center Houston TX USA; ^2^ Department of Biostatistics University of Texas M.D. Anderson Cancer Center Houston TX USA; ^3^ Department of Biostatistics Cleveland Clinic Cleveland OH USA

**Keywords:** Cancer—surgery, estimated glomerular filtration rate, renal function, ureter, ureteroureterostomy

## Abstract

**Objectives:**

To evaluate the long‐term renal function outcomes after ureteroureterostomy (UU) in patients undergoing multi‐organ resection for non‐urothelial cancers. The secondary aim was to examine the length of ureteric defect that can be successfully bridged with UU.

**Patients and methods:**

We retrospectively reviewed the charts of patients who underwent UU between 1995 and 2012 at our institution. Renal imaging studies performed before and after UU were used to determine whether hydronephrosis was present. Renal function was assessed by comparing estimated glomerular filtration rate (eGFR) before and at the last follow‐up after UU.

**Results:**

Nineteen patients underwent UU during multi‐organ resection for non‐urothelial cancers. Median follow‐up time was 62 months. Overall, UU had a high success rate, with one patient (5.2%) developing progressive hydronephrosis with a >20% drop in eGFR from baseline due to UU failure. Four additional patients developed progressive hydronephrosis due to cancer recurrence involving the UU. There were no statistically significant differences between pre‐ and post‐UU eGFR in these patient cohort. All patients with a ureteric defect of ≤5 cm underwent successful reconstruction.

**Conclusions:**

UU maintains long‐term renal function in the majority of patients undergoing multi‐organ resection for non‐urothelial cancers and can be successfully utilized if the resected ureteric length is ≤5 cm.

## 1 INTRODUCTION

Resection of a segment of one or both ureters is sometimes necessary to adequately remove advanced pelvic or retroperitoneal cancers. Depending on the length and location of the resected segment, a variety of ureteric reconstruction options are available.^1^ If the resected segment of the ureter is mid to distal, then ureteroneocystostomy with or without an elongation procedure (psoas hitch or Boari flap) is simple and highly successful.^2^ However, if a relatively short section of the mid or upper ureter is resected, then ureteroureterostomy (UU) may be feasible, particularly in cases in which the distal ureter is viable. Although UU is considered when the ureteric defect is short; the length of ureteric defect that can be bridged with UU including aggressive nephropexy has not been well studied. Some cancers may displace and stretch the ureter allowing a longer ureteric defect to be successfully bridged with UU.

There are very limited reports in the literature regarding renal functional outcomes of UU reconstruction during multi‐organ resection for cancer^3^, ^4^, ^5^, ^6^, ^7^(Table 1). Most of the earlier studies include few patients and failure of the UU based on the development of stricture or hydronephrosis is reported in 27%‐100% of patients.^3^, ^4^, ^5^, ^6^, ^7^ One of the largest series, by Fry et al^8^ included two patients that died of urine leakage and sepsis in the postoperative period.^3^ Only one of the earlier reports examine renal functional outcomes using estimated glomerular filtration rates based on serum creatinine measurements. Although there are no standardized or agreed upon definitions of success following UU reconstruction, successful reconstruction would include stable renal function and the absence of progressive hydronephrosis or stricture on subsequent imaging studies.

**TABLE 1:** Prior studies of ureteroureterostomy performed during multi‐organ resection for cancer

Study	N	Diagnosis	Follow‐up	Evaluation of GFR	Definition of success	Complications	Failure of UU
Hoffman, 2006^4^	7	Ovarian cancer, cervical cancer, or endometrial cancer	(1) Lost to follow‐up; (1) 9 months; (1) 20 months; (2) 22 months; (2) strictured	No	No	2 of 7 had strictures on removal of stent	2 of 7 (28%)
Fry, 1983^3^	11	Mixed population benign and cancer (ovarian, colorectal, or endometrial)	(4) Long‐term satisfactory 2‐21 years; (4) Lost to follow‐up after 1 yr; (2) Short term‐failure; (1) Long‐term failure	No	No	(2) Short‐term failures; each with leakage, sepsis, and death	3 of 11 (27%)
						(1) Long‐term failure due to severe hydronephrosis because of stenosis of the anastomosis	
Chien‐ Min Han, 2011^5^	2	Endometrial and cervical cancer	33 and 57 months	Yes	No	(1) Stricture; (1) Hydronephrosis	2 of 2 (100%)
Berek,1982^6^	2	Ovarian Cancer	NA	No	No	NA	NA
Stocchi,2006^7^	6	Rectal Cancer	At least 2 years or until death	No	No	NA	NA
Morkavuk (2020)^8^	2	Gynecologic, colorectal, and three retroperitoneal sarcomas (cytoreductive surgery with hyperthermic intraperitoneal chemotherapy HIPEC)	Mean 11.6 Months	No	No	(1) Urine leak	N/A
Present Study	19	Retroperitoneal sarcoma (5), colon cancer (4), testicular cancer (3), appendiceal cancer (2), endometrial adenocarcinoma (2), lymphoma (2), and ovarian cancer (1)	Median 62 months; Range (9‐180 months)	Yes	Yes	(1) Urine leak, subsequent anastomotic stricture with hydronephrosis, and reduced kidney function	1 of 19 (5.2%)

The aim of our study is to evaluate long‐term renal functional outcomes following complex multi‐organ resection for non‐urothelial cancers with UU reconstruction. A secondary aim was to examine the length of ureteric defect that can be successfully bridged with UU.

## 2 PATIENTS AND METHODS

A research protocol to evaluate renal function outcomes after a variety of ureteric reconstructions including UU was approved by the Institutional Review Board at the University of Texas MD Anderson Cancer Center. We conducted a retrospective chart review of all patients who underwent UU for a complete ureteric transection between 1995 and 2012. All patients in this study were undergoing complex cancer surgery for non‐urothelial cancers. Patients who had other modalities of diversions or had a UU for urothelial cancers were excluded. As these patients underwent complex cancer surgery involving multidisciplinary surgical approach, all patients were counseled pre‐operatively about all the available reconstruction options, the risk, and benefits by the treating urologist. However, the decision to perform a UU reconstruction vs other options was made intraoperatively by the treating urologist. In all patients undergoing UU, the proximal and distal ureters were spatulated and anastomosed end to end with absorbable sutures over a ureteric stent. Nephropexy was utilized as needed. The duration of ureteric stenting was at the discretion of the treating surgeon.

Patients medical records were reviewed in detail for demographic data, oncologic information, and renal function including anastomotic patency, stricture development, and evidence of progressive hydronephrosis on serial imaging including CT scans, MRI’s, intravenous pyelography, abdominal ultrasound tests, or obstruction on nuclear renal scan. The failure of UU was defined as the progression or development of new hydronephrosis on serial imaging and >20% drop in eGFR after the first 3 weeks of follow‐up. Patient's imaging prior to UU was assessed for baseline hydronephrosis of the index kidney and each postoperative imaging test including cancer surveillance imaging were reviewed to determine whether there was progression of hydronephrosis.

The serum creatinine was assessed prior to and following the UU with calculation of estimated glomerular filtration rate (eGFR) based on the Cockgraft‐Gault equation and adjusted for patient race. The drop in eGFR of 20% below baseline parameter for failure of the UU was selected based on the fact that the National Kidney Foundation Kidney Disease Outcomes Quality Initiative (KDOQI) staging of CKD has a reduction between 15 and 30 ml/min//1.73 m^2^ in GFR between CKD staging levels^9^ and the fact that many nephrologists consider a drop in eGFR of 20% below baseline to be a significant concern and possible indication for closer monitoring or further investigation. Creatinine values within the first 3 weeks immediately following surgery were not considered in the analysis since most of the patients had a transient elevation in baseline creatinine with the insult of surgery and since we were interested in assessing long‐term functional results of the UU reconstruction.

Patients who developed recurrence of cancer involving the UU were not considered as a primary failure of the UU, but rather a manifestation of aggressive biology of a high‐grade neoplasm and were censored at the time obstruction of the UU from cancer was first seen on imaging. In these patients, who developed worsening hydronephrosis due to recurrence of cancer involving the UU, the serum creatinine of the visit prior to the recurrence was considered as the final post‐UU creatinine. The length of ureteric defect was determined by the pathologist's measurement of the length of resected ureter in the pathology report or based on the surgeon's description of the length of ureteric defect in the operative report if no pathologic measurement was available. Complications from UU were reported according to the Clavien‐Dindo classification.

The two sample *t* test was used to compare the baseline and final post‐UU eGFR and *p* value of <.05 was considered statistically significant. Kaplan‐Meier curves were used for failure‐free survival analyses. SPSS statistical software (version 26.0) was used for statistical analysis.

## 3 RESULTS

A total of 32 patients undergoing UU were identified; no follow‐up information was available for 8 patients leaving 24 evaluable. Of these, 5 patients were also excluded as they had partial ureteric transection and did not have a ureteric segment excised, leaving 19 patients for the final analysis. Patient characteristics including age, extent of prior therapy for cancer, side and length of ureteric defect, development of progressive hydronephrosis, follow‐up, and oncologic status are shown in Table 2. The patients’ median age was 57 years (range 23‐68 years). The median follow‐up time was 62 months (range 9‐180 months). Short follow‐up times in some patients reflected rapid disease progression and cancer death.

**TABLE 2:** Patient characteristics, prior therapy, surgical parameters, cancer progression, and UU‐success in 19 patients undergoing UU

Patient	Sex	Age Yrs	Diagnosis	Prior radiation	Prior chemo	Prior surgery	Index kidney	Length (cm)	Location ureter	UU success at end of follow‐up	Progression of hydro	Disease relapse	Follow‐up (months)	Death from cancer
1	M	44	Hodgkin's disease	Y			L	1	mid	Y			180	
2	F	67	Retroperitoneal sarcoma	Y	Y		L	5.6^b^	mid	Y		Y	53	Y
3	M	25	Lymphoma			Y	L	1.7^b^	mid	Y			127	
4	F	57	Peritoneal carinomatosis & pseudomyxoma peritonei from appendiceal mucinous adenocarcinoma		Y	Y	L	1.5	lower	Y			140	
5	M	68	Retroperitoneal sarcoma				L	3	mid	Y			124	
6	F	49	Retroperitoneal sarcoma		Y	Y	L	5^b^	mid	Y		Y	122	
7	F	52	Recurrent Leiomyosarcoma of the retroperitoneum		Y	Y	R	2	mid	Y	Y	Y	75	Y
8	F	63	Metastatic adenocarcinoma of the appendix		Y		R	2.3^b^	mid	Y^a^		Y	9	Y
9	F	64	Endometrial cancer				L	0.5^b^	mid	Y			81	
10	M	45	Colon Cancer		Y	Y	R	3	mid	Y			95	
11	M	23	Nonseminomatous testicular cancer		Y	Y	L	5.2^b^	mid	Y^a^		Y	9	Y
12	F	62	Recurrent colon carcinoma	Y	Y	Y	L	3	mid	Y	Y	Y	74	
13	F	60	Granulosa cell cancer		Y	Y	L	4	mid	Y		Y	62	Y
14	F	65	Endometrioid Adenocarcinoma				L	1	mid	Y			52	
15	M	43	Locally recurrent colorectal carcinoma		Y	Y	R	3	mid	Y	Y	Y	15	Y
16	M	65	Colo‐rectal carcinoma				R	4^b^	mid	Y	Y	Y	22	Y
17	M	24	Metastatic testicular cancer		Y	Y	R	2.5^b^	mid	Y			39	
18	M	31	Nonseminomatous testicular cancer		Y	Y	R	4.2^b^	mid	Y			30	
19	M	66	Retroperitoneal sarcoma	Y	Y		L	8.5^b^	mid	^a^	Y	Y	27	

Note

Code: Y = yes.

^a^ indicates chronic ureteric stenting with progressive cancer in two patients and stricture of the UU in one patient.

^b^ denotes formal pathologic measurement of the excised ureteric length.

The type of neoplasm included sarcoma (five patients), colon cancer (four patients), testicular cancer (three patients), appendiceal cancer (two patients), endometrial cancer (two patients), lymphoma (two patients), and ovarian cancer (one patient). The patients were heavily pretreated for cancer including prior radiation therapy in 4 patients, prior chemotherapy in 13 patients, and prior surgery in 11 patients. Six patients (31.6%) had baseline hydronephrosis of the index kidney prior to the UU surgery. The ureteric defect was located in the mid ureter in 18 patients, and the lower ureter in 1 patient. Twelve of the patients underwent left‐sided UU and seven right‐sided UU. Four patients had radiation post‐UU. The length of ureteric defect was ≥4 cm in seven patients.

**FIGURE 1:**
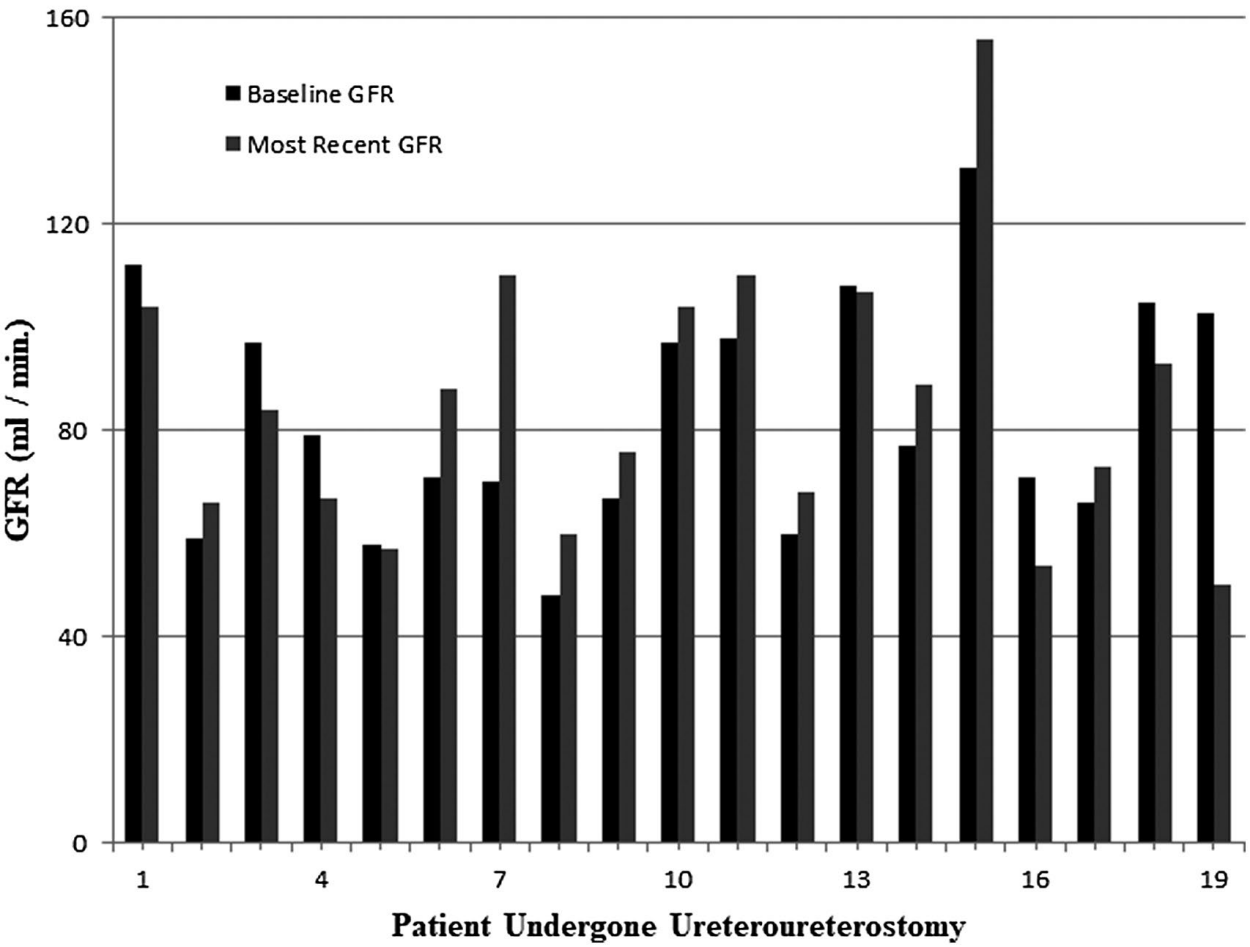
Baseline and last follow‐up eGFR in 24 patients undergoing UU. Baseline GFR is denoted in black and last follow‐up GFR in gray

**FIGURE 2:**
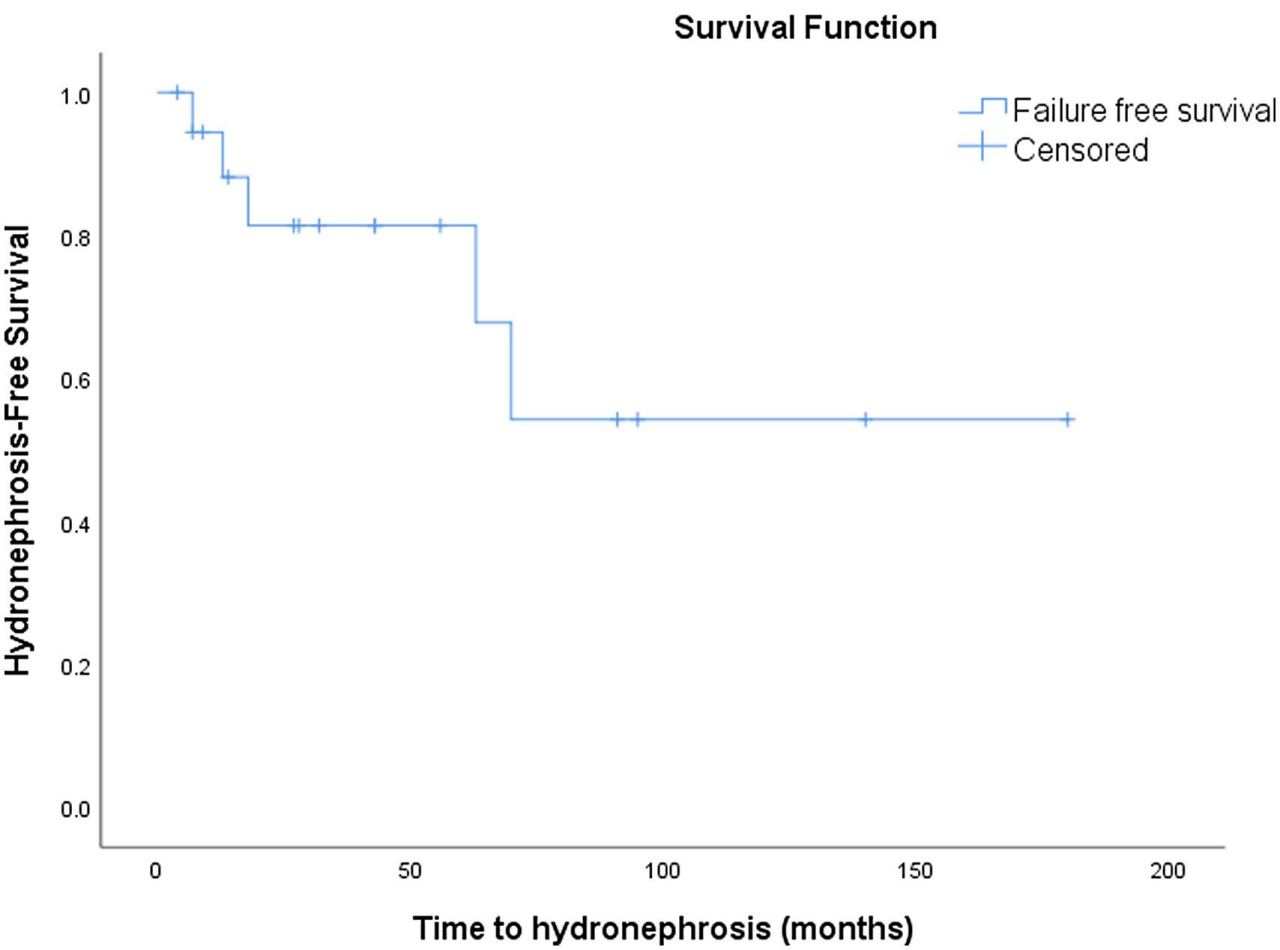
Kaplan‐Meier graph demonstrating new or progressive hydronephrosis‐free survival in patients undergoing UU during multi‐organ resection (including patients who had worsening hydronephrosis due to progression of disease). Patients who were lost to follow‐up or died were censored

All patients had pre‐ and post‐UU eGFR information. The average eGFR at baseline and last follow‐up were 81.2 mL/min and 83.1 mL/min, respectively (Figure 1). There was no significant difference between baseline and last follow‐up eGFR (*P* = .54). Seventeen patients had pre‐ and post‐UU CT scans. Post‐UU imaging included a total of 88 CT scans (mean 4.6 per patient, range 0‐13 per patient), 9 diuretic renal scans, 8 ultrasounds, and 2 MRI exams. Overall, UU had a high success rate in these patients with only one failure of the reconstruction (5.2%) (Figure 2A). Although a total of five patients developed progressive hydronephrosis on serial imaging, of which four patients had recurrence of cancer involving the UU (Figure 2B). The patient who had primary failure of the UU had a long mid‐ureteric stricture (8.5cm) developed a urine leak and subsequent stricture with progressive hydronephrosis on imaging and *a* > 20% drop in eGFR from baseline. The longest successfully reconstructed ureteric defect was 5.6cm in the mid ureter.

Relapse of cancer occurred in 10 patients of which 7 have died (median time to death: 22 months, range 9‐75 months). Two patients had chronic indwelling ureteric stents after UU due to cancer progression.

There were a total of nine surgical complications in eight patients related to the UU. Three patients had bladder irritation or pain from the ureteric stent (Clavien grade one) and one patient had a urinary tract infection (Clavien grade two). There were four patients (20.5%) with Clavien ≥3 complications post‐UU (Table 3).

**TABLE 3:** Surgical complications (Clavien grade ≥3) of UU in 19 patients

Patient	Complication	Clavien grade
6	Ureteric stent migration requiring ureteroscopic removal	3B
9	Temporary edema and obstruction of UU during and immediately following postoperative radiation requiring separate nephrostomy tube and ureteric stent	3A, 3B
18	Ureteric stent migration requiring ureteroscopic removal	3B
19	Failure (obstruction) of UU requiring ureteric stent	3B

## 4 DISCUSSION

We present the long‐term renal functional outcomes using a combination of eGFR and imaging after UU in a series of non‐urothelial cancer patients undergoing multi‐organ resection. Renal preservation is essential in cancer patients undergoing multi‐organ resection as majority require additional systemic therapies. Chronic kidney disease (CKD), a graded and independent risk factor for substantial morbidity and death has been found to complicate the treatment of cancer patients.^10^ While, all surgical options should be considered to optimize renal preservation in these patients, UU is rarely utilized as a re‐constructive option of the ureter, mainly owing to the high rates of complications seen in small studies (Table 1). However, UU is successfully utilized at renal transplant with a meta‐analysis demonstrating no difference in the overall rates of complications between UU and uretero­neocystostomy, with stricture, obstruction and stone formation being the more common complications associated with UU.^11^ In contrast to the other studies, only one patient in our study (5.2%) had failure of the UU as defined by *a* > 20% drop in eGFR from baseline and progressive hydronephrosis from anastomotic stricture and obstruction of the UU. We found that differences in patients eGFR’s before and after UU were not significant; this finding suggests that long‐term renal function is maintained in the majority of patients who undergo UU. While not statistically significant, some patients experienced an increase in eGFR following UU, possibly due to improved index kidney function after relief of obstruction in these patients.

Successful UU during multi‐organ resection requires a meticulous surgical technique and can be used in select cases.^1^ The anastomosis needs to be tension‐free and aggressive mobilization of the kidney should be considered if those cases where tension may exist. The distal ureter must be viable with reasonable blood supply. EAU guidelines recommend UU for proximal mid‐ureteric injuries involving a ureteric segment of <2‐3 cm.^2^ The longest ureteric defect to be successfully reconstructed in our series was 5.6 cm and the single patient who experienced failure of the UU had a mid‐ureteric defect of 8.5 cm. Thus our data indicate that UU can be utilized successfully if the ureteric defect is ≤5 cm when combined with aggressive nephropexy (when feasible) in this cohort of patients undergoing multi‐organ resection.

Four patients (21%) undergoing UU developed had Clavien 3 complications related to the UU reconstruction in our series. These complication rates are in keeping with other series where different reconstruction options for the ureter were utilized.^8^, ^12^ In the series by Federico et al, 26.1% of patients experienced early and late complications with 30.4% developing severe urological complications.^12^ Majority of the late complications (64.2%) in that series included urinary leakage (19.5%), hydronephrosis (6.5%), and 4.3% of unilateral renal impairment requiring nephrectomy.^12^ In the series by Morkavuk reported on a series of 20 patients undergoing cytoreductive surgery with hyperthermic intraperitoneal chemotherapy (HIPEC) with segmental ureteric resection with reconstruction using a different reconstructive options including UU, Trans‐ureteroureterostomy, ureteroneocystostomy, Boari flap, and ureterosigmoidostomy.^8^ They reported grade 3 or more complication rates in 35% of patients including early anastomosis leakage in five patients (10%) and late anastomosis stricture in three patients (15%).

Patients undergoing UU during multi‐organ resection have cancers with heterogeneous biology and are often undergoing “desperation” surgery. Therefore, our primary goal was not to assess oncologic outcomes, but rather success of the UU reconstruction. We also included patients who developed cancer recurrence involving the UU with progressive hydronephrosis. Each of these patients developed significant recurrence in the abdomen or pelvis due to aggressive tumor biology and three of these four patients died of progressive malignancy. In order to reduce bias and identify the actual stricture rates associated with UU, we utilized the eGFR values just prior to cancer recurrence in these patients.

The main limitations of our paper are its retrospective nature, limited number of patients and procedures performed at a high volume center which may not be generalizable. However, our report is the largest‐to‐date of patients undergoing UU during multi‐organ resection of cancer (Table 1). An additional strength of our paper is that all patients had baseline and post‐UU eGFR information and post‐UU imaging with long‐term data available for the majority due to ongoing cancer surveillance allowing us to demonstrate durable success in the majority of patients. While we attempted to minimize bias by inclusion of the patients who progressed, this may have also affected our results. Additionally we evaluated the preservation of the global renal function which could have been affected by and other factors such as postoperative chemotherapy use. Furthermore, not all patients had postoperative renograms of the index kidney, therefore, the function of the individual kidney could not be assessed. Although patients underwent different diversions during this period, the primary aim of this study was to demonstrate the efficacy of UU an option by specifically evaluating long‐term renal function in patients who underwent UU reconstruction. Only 10 out of the 19 (52.6%) patients had formal pathologic measurement of the excised ureteric length which may have introduced a subjectivity in length of the excised segment of ureter in the smaller defects.

## 5 CONCLUSIONS

Long‐term renal function following UU is maintained in the majority of patients undergoing multi‐organ resection for non‐urothelial cancers. The UU can be utilized successfully if the ureteric defect is ≤5 cm when combined with aggressive nephropexy, in carefully selected patients. Larger prospective studies are needed to investigate the utility of UU in patients undergoing multi‐organ resection for cancer.
